# First metatarsophalangeal joint arthrodesis/fusion: a systematic review of modern fixation techniques

**DOI:** 10.1186/s13047-022-00540-9

**Published:** 2022-04-26

**Authors:** Yang S. Kang, Andy Bridgen

**Affiliations:** Department of Podiatry, School of Human and Health Sciences, University of Huddersfield, Huddersfield, UK

**Keywords:** Arthrodesis, Fusion, First metatarsophalangeal joint, Hallux abducto valgus, Hallux rigidus, Systematic review

## Abstract

**Background:**

First metatarsophalangeal joint arthrodesis is commonly performed for symptomatic end-stage hallux rigidus. It has been postulated to produce good results in the literature. Various fixation techniques offer differences in union rates, complications and functional outcomes, stirring debates about which produces the best outcomes for patients. Therefore, this review aims to synthesise and compare the outcomes of modern fixation techniques used for first metatarsophalangeal joint (FMPJ) arthrodesis.

**Methods:**

The electronic database searched were PubMed, CINAHL, Cochrane Library, and Google Scholar. The critical appraisal skills programme tool for cohort study was used. The interventions consisted of screw(s), plate(s), and staple(s). Studies comprising outdated fixation techniques such as suture, metallic wire, external fixation, Rush rods or Steinmann pins were excluded. Participants were adults over 18 years, undergoing FMPJ arthrodesis in the UK. Studies with the population consisting primarily of revision cases, patients with rheumatoid arthritis or diabetes were excluded.

**Results:**

Seven UK studies included 277 feet and a 95.7% overall union rate at a mean union time of 83.5 days. Staples had the highest union rate of 98.2% at mean union time of 84 days, followed by plates (95.2%, 92 days), and finally screws (94.9%, 71 days). The overall complication incidence is 5.8%. All of the fixation techniques produced good functional outcomes postoperatively.

**Conclusions:**

Whilst staple techniques showed the highest union rate, plating techniques are preferable over screws or staples for better results across several outcome measures, including reduced complication incidence, stability, early ambulation, and good functional outcome. The Manchester-Oxford Foot Questionnaire and EuroQol-5Dimensional are recommended as measurement tools to assess functional outcomes following FMPJ arthrodesis.

**Supplementary Information:**

The online version contains supplementary material available at 10.1186/s13047-022-00540-9.

## Background

First metatarsophalangeal joint (FMPJ) arthrodesis is a commonly performed procedure for symptomatic end-stage hallux rigidus (grades 3 and 4) [1, 2]. The procedure can also be considered in selected cases of severe hallux abducto valgus (HAV) deformity associated with degenerative joint disease and as a salvage option for previously failed first ray procedures [3]. Nix et al. [4] found HAV occurrence in 23% of people aged 18 to 65 and 35.7% in those over 65. In the United Kingdom (UK), Roddy et al. [5] observed a 16.7% overall population prevalence of symptomatic foot osteoarthritis in adults over 50 years and 7.8% specifically for hallux rigidus. Furthermore, Morgan et al. [6] reported an increasing foot osteoarthritis trend and estimated a significant increase in hallux rigidus incidence between 2000/2001 and 2017/2018 in England.

The UK provides public and free healthcare coverage, known as the National Health Service (NHS), to all permanent residents at the point of need and is funded primarily through general taxation [7]. A potential drawback from this publicly-funded universal healthcare system is that each provider receives a limited budget which may not necessarily cover demand, increasing financial pressures [8]. Furthermore, the national initiative ‘Getting it Right First Time’ project identified an increasing requirement for orthopaedic treatment nationally, urging attempts to identify and administer effective interventions that result in cost savings, minimal complications and optimal patient outcomes [9]. Therefore, it is essential to take a methodical approach by first determining which fixation technique for FMPJ arthrodesis can best address the initiative. Treadwell [10] described that the best fixation technique for FMPJ arthrodesis should achieve a high union rate with minimal fixation-related complications, absolute stability and early ambulation, all of which reflects the AO fixation principles [11]. Whilst it seems easy to detail the best fixation technique, the choice may not be clear despite the abundance of literature available. The union rate of FMPJ arthrodesis is commonly reported in the literature as approximately 90% [12]; however, a review of these reports revealed the inclusion of outdated fixation techniques and patients mainly with rheumatoid arthritis and salvage procedures, allowing inaccurate reflection of the actual union rate. Individual studies also have different interpretations of complications, as some included procedure-related (i.e., metatarsalgia, hallux varus) rather than fixation-related complications (i.e., metalwork complications, malunion, revision) in their findings.

Clutton [13] first described FMPJ arthrodesis as a treatment for severe HAV using an ivory peg as a fixation device but was later popularised by Thompson and McElvenny [14] for the treatment of end-stage hallux rigidus. Since the 1980s, fixation techniques have developed from suture, metallic wire, external fixation, Rush rods, and Steinmann pins to modern-day screws, plating systems, and staples [15]. As a result, modern fixation techniques have been postulated to promote fusion and functional outcomes whilst reducing fixation-related complications [16]. Roukis [15] performed a systematic review of 2818 FMPJ arthrodesis procedures finding an overall union rate of 94.6% and complication incidence of 6.1% for malunion and 8.5% for metalwork removal. Korim et al. [17] also conducted a systematic review of 2059 FMPJ arthrodesis procedures obtaining an overall union rate of 93.5% and a 7.5% complication rate, consisting of only metalwork removal. Both systematic reviews focused only on union rate and complications, disregarding patients’ functional outcomes. Furthermore, only the overall union rate of all the techniques combined was considered, rather than individually. Consequently, there is no consensus on which fixation technique is best for FMPJ arthrodesis, making it challenging for providers to identify priorities and allocate their resources accordingly.

In essence, union rates, complication incidences and functional outcomes are essential determinants of successful FMPJ arthrodesis. Also, the choice of fixation technique should produce good postoperative outcomes and reflect fixation principles established by the renowned AO Group [11]. As there is a high prevalence of HAV and an increase in hallux rigidus incidence in the UK, FMPJ arthrodesis has already been a standard and extensively performed procedure [18]. Therefore, even the slightest improvement in union rate and functional outcomes with a lowering complication incidence would significantly impact the overall cost of health services and patient satisfaction. Accordingly, this systematic review aims to synthesise and compare modern fixation techniques used for FMPJ arthrodesis in the UK.

## Methods

This systematic review was reported in accordance with the Preferred Reporting Items for Systematic Reviews and Meta-Analyses (PRISMA) guidelines [19]. See Additional file 1.

### Search strategy

The electronic databases searched were PubMed, CINAHL, and Cochrane Library. The date limit was set from January 2010 to April 2021. Google Scholar was also utilised to capture any studies that may have been missed out. The following search terms ‘arthrodesis’, ‘first metatarsophalangeal joint’, ‘staple’, ‘screw’, ‘plate’, and related synonyms were formulated, combining each search term using Boolean operators (‘AND’, ‘OR’ and ‘*’) as appropriate. Subject headings were exploded where appropriate according to each database. See Additional file 2.

### Eligibility criteria

The eligible study designs were randomised controlled trials (RCTs), cohort studies and case series. The articles included were in the English language, published within peer-reviewed journals, and consisted of at least 20 patients and feet. Cadaveric and biomechanical studies were excluded as functional outcomes can only be assessed through human participants. Participants in this review were adult males and females over 18 years. Only studies conducted in the UK were eligible as this review pertains to the UK health system. Studies with the population consisting primarily of revision cases, patients with rheumatoid arthritis or diabetes were excluded. The studies’ interventions included in this review were fixation techniques involving screw(s), plate(s), and staple(s). Studies comprising outdated fixation techniques such as suture, metallic wire, external fixation, biodegradable or Rush rods, and Steinmann pins were excluded. The studies included in this review comprised all three union rates, complications and functional outcomes. The unions were radiographically validated, visually confirming trabecular bridging of bone. The complications assessed in this review were fixation-related, not procedure-related. Fixation-related complications included malunion, delayed union, soft tissue damage, need for a revision procedure, and metalwork complications such as removal and difficulty inserting metalwork. Procedure-related complications were metatarsalgia, wound infections, hallux varus, recurrence, and non-specific idiopathic pain. The functional outcomes of studies were measured quantitatively using questionnaires and scoring systems involving numbers rather than exploratory.

### Study selection and data collection process

The titles and abstracts identified through the electronic database and other searches were exported to EndNote X9 software [20], removing duplicates. Similar articles published in different journals were also removed. Each record was assessed for inclusion according to the predefined eligibility criteria. Next, the full-text articles were retrieved to determine the eligibility for qualitative synthesis. Two independent reviewers screened each stage, and any discrepancies were resolved through discussion.

### Quality assessment

The Critical Appraisal Skills Programme (CASP) [21] tool was used to assess the quality of the studies. Two independent reviewers conducted the quality assessment, and a consensus was reached to assess methodology quality, whereby no concern (= all ‘yes’), minor concerns (= most ‘yes’ and one ‘no’ or ‘can’t tell’), and major concerns (= more than one ‘no’ and ‘can’t tell’). Any disputes were resolved through discussion.

### Data synthesis

One review author (YSK) systematically extracted data from all the included studies using a piloted data extraction form created in Microsoft Word and was crossed-checked by the second review author (AB). Any disagreements were resolved through discussion. The following data was extracted and categorised accordingly as screw, plate, and staple: year, study design, setting and period, sample size, patient demographic, indication for surgery, mean follow-up and the ranges, intervention type, bone union outcome, mean union time, fixation-related complications, functional outcome tool.

The total number of patients and feet were calculated within each category. The union rate and complication incidence were determined using the formula $\frac{\mathrm{Total}\ \mathrm{bone}\ \mathrm{union}}{\mathrm{Total}\ \mathrm{feet}}\times 100\%$ and $\frac{\mathrm{Total}\ \mathrm{complication}}{\mathrm{Total}\ \mathrm{feet}}\times 100\%$, respectively. The best fixation technique was determined by the technique which closely adheres to fixation principles and shows the highest union rate, lowest fixation-related complication, and significant improvements in functional outcomes pre to postoperatively.

## Results

### Search results

**Fig. 1:**
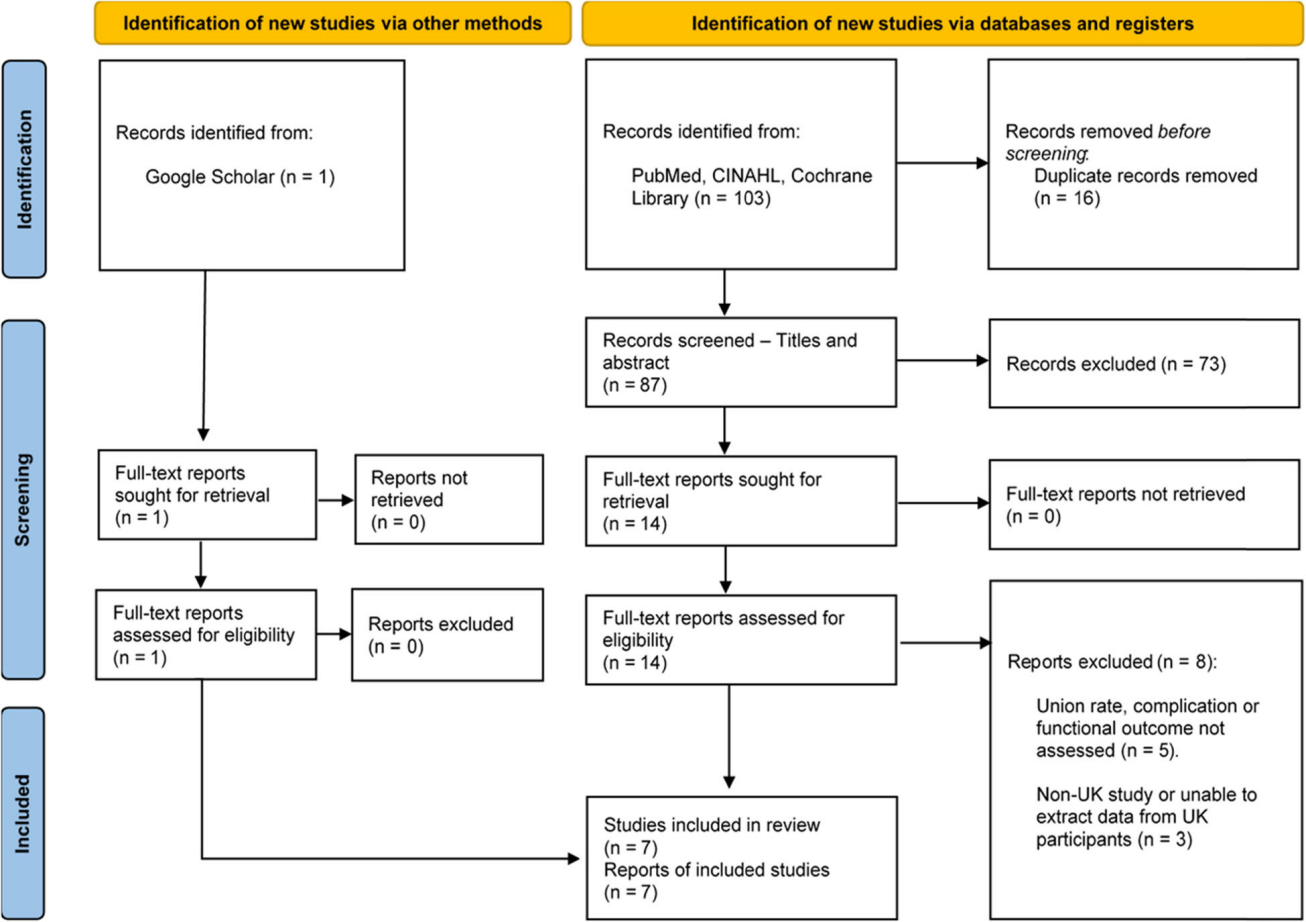
PRISMA flow diagram

The initial search resulted in 104 articles. Of these, 16 duplicate articles were removed, and a further 73 were excluded at the abstract and title screening stage. Next, 15 full-text articles were assessed for eligibility, of which eight were rejected [22–29]. Ultimately, seven UK studies were included in this systematic review. The PRISMA flow diagram (Fig. 1) was utilised to depict the flow of studies selected through different phases of this systematic review, and the citations of articles excluded at the full-text screening stage, with reasons, are available in Additional file 3.

### Characteristics of included studies

The eligible studies consisted of one prospective and six retrospective cohort study designs conducted in hospitals in England, Wales and Scotland. Three studies used screws, three used plates, and one used staples as fixation techniques for FMPJ arthrodesis. Mohammed & Gadgil [30] utilised a two-crossed screw method using BOLD® cannulated, self-drilling, self-tapping screws. Two studies [31, 32] utilised an Intra-Osseous FIXation (IOFIX) device that accommodates a lag screw to pass at a 60° angle across the fusion site. Fazal et al. [33] utilised two orthogonal two-holed locking plates with two locking screws in each plate. Latif et al. [34] utilised an Anchorage Stryker® cross plate, a low profile locking plate that allows an interfragmentary lag screw insertion through the counterbore within the plate’s shaft. Marudanayagam and Appan [35] utilised a low profile Fyxis plate prebent to 5° dorsiflexion and an oblong hole to insert an interfragmentary screw. Challagundla et al. [36] utilised two Memory staples placed in an orthogonal orientation across the fusion site. The complete characteristics of the studies were presented in four tables: Tables 1, 2, 3 and 4.

**Table 1:** Characteristics of each study

Author	Study design	Number of patients (No. of feet)	Mean age (years, range)	Mean follow-up (months, range)	Mean time to union (days, range)	Intervention	Bone union outcome (No. of feet)	Functional outcome tool
Patel et al. [31]	Retrospective Cohort	52 (54)	64 (36–78)	14.2 (12.0–23.0)	61 (93–201)	Screw	96.3% (52/54)	MOXFQ
Singhal et al. [32]	Retrospective Cohort	21 (21)	63 (47–81)	28.0 (13.4–48.2)	81 (40–222)	Screw	95.0% (20/21)	MOXFQ
Mohammed & Gadgil [30]	Retrospective Cohort	21 (23)	60 (31–84)	17.0 (6.0–34.0)	Unknown	Screw	91.0% (21/23)	AOFAS-HMI
Latif et al. [34]	Prospective Cohort	36 (38)	56 (20–74)	15.0 (3.0–42.1)	84 (42–112)	Plate	100.0% (38/38)	MOXFQ, EQ-5D
Fazal et al. [33]	Retrospective Cohort	26 (32)	64 (40–78)	49.0 (40.0–62.0)	94 (42–183)	Plate	84.3% (27/32)	AOFAS, FADI
Marudanayagam & Appan [35]	Retrospective Cohort	51 (54)	58 (28–74)	14.8 (12.0–20.0)	97 (91–183)	Plate	98.1% (53/54)	AOFAS
Challagundla et al. [36]	Retrospective Cohort	50 (55)	63 (41–77)	38.0 (12.0–73.0)	84 (Unknown)	Staple	98.2% (54/55)	FAAM ADL
**Total/Mean**	**-**	**257 (277)**	**61** **(20–84)**	**25.1** **(6.0–73.0)**	**82.3**^**a**^ **(40–222)**	**-**	**95.7%** **(265/277)**	**-**

*MOXFQ* Manchester-Oxford Foot Questionnaire, *AOFAS-HMI* American Orthopaedic Foot and Ankle Society-Hallux Metatarsophalangeal Interphalangeal, *EQ-5D* EuroQol–5 Dimensional, *FADI* Foot and Ankle Disability Index, *FAAM ADL* Foot and Ankle Mobility Measure (Activities of Daily Living)

^a^ Mohammed & Gadgil [30] excluded from mean union time, as it is unknown

**Table 2:** Characteristics of each study (Sex)

Author	Sex and number of patients	
	**Male**	**Female**
Patel et al. [31]	14	38
Singhal et al. [32]	4	17
Mohammed & Gadgil [30]	9	12
*Latif et al. [34]	Unknown	Unknown
Fazal et al. [33]	3	23
Marudanayagam & Appan [35]	12	39
Challagundla et al. [36]	14	36
**Total**	**56**^**a**^	**165**^**a**^

^a^Latif et al. [34] did not disclose their sex proportion and is therefore excluded from the total count.

**Table 3:** Characteristics of each study (Fixation related complications)

Author	No. of feet	Fixation related complications				
		**Revision surgery**	**Metalwork complications**	**Delayed union**	**Soft tissue damage**	**Total**
Patel et al. [31]	54	1	3	-	-	**4**
Singhal et al. [32]	21	-	-	1	-	**1**
Mohammed & Gadgil [30]	23	1	-	-	-	**1**
Latif et al. [34]	38	-	1	-	-	**1**
Fazal et al. [33]	32	-	1	-	-	**1**
Marudanayagam & Appan [35]	54	1	-	-	2	**3**
Challagundla et al. [36]	55	-	1	3	1	**5**
**Total**	**277**	**1.08%**	**2.17%**	**1.44%**	**1.08%**	**5.8%** **(16/277)**

**Table 4:** Characteristics of each study (Indication for surgery)

Author	Indications for surgery and number of feet				
	**Severe hallux abducto valgus**	**End-stage hallux rigidus**	**Revision surgery**	**Rheumatoid arthritis**	**Total**
Patel et al. [31]	8	44	-	2	**54**
^a^Singhal et al. [32]	Unknown	Unknown	Unknown	Unknown	Unknown
Mohammed & Gadgil [30]	9	7	3	4	**23**
Latif et al. [34]	-	31	-	7	**38**
Fazal et al. [33]	-	32	-	-	**32**
Marudanayagam & Appan [35]	8	38	-	8	**54**
Challagundla et al. [36]	6	41	8	-	**55**
**Total**	**31**	**193**	**11**	**21**	**256**^**a**^

^a^Singhal et al. [32] did not provide their indications for surgery and is therefore excluded from the total count

### Quality assessment of included studies

Five studies [30, 31, 34–36] had major concerns relating to lack of clarity when reporting definitions, patient recruitment, missing data, and no blinding of assessors. Two studies [32, 33] had minor concerns because confounding factors were not accounted for by performing any stratification, multivariate models or regression analyses. All studies demonstrated concerns for quality because these studies’ nature were descriptive cohort studies, in which a comparator or a control group is lacking, and efforts to control confounding were absent. The overall assessment of each study was presented in Table 5.

**Table 5:** Quality assessment for included studies. Q1 to Q11 represents the questions in the CASP tool for cohort studies

	Mohammed [30]	Marudanayagam [35]	Fazal [33]	Singhal [32]	Latif [34]	Patel [31]	Challagundla [36]
**Q1**	Yes	Yes	Yes	Yes	Yes	Yes	Yes
**Q2**	Yes	Can’t tell	Yes	Yes	Yes	Yes	Yes
**Q3**	Yes	Yes	Yes	Yes	Yes	Yes	Yes
**Q4**	No	No	Yes	Yes	Yes	Yes	Can’t tell
**Q5**	No	No	Yes	Yes	No	No	No
**Q6**	No	No	No	No	No	No	No
**Q7**	No	Yes	Yes	Yes	No	No	No
**Q8**	Yes	Yes	Yes	Yes	Yes	Yes	Yes
**Q9**	No	No	Yes	Yes	Yes	Yes	Can’t tell
**Q10**	Yes	Yes	Yes	Yes	Yes	Yes	Yes
**Q11**	Yes	Yes	Yes	Yes	Yes	Yes	Yes
**Overall** **assessment**	Major concern	Major concern	Minor concern	Minor concern	Major concern	Major concern	Major concern

1. Did the study address a clearly focused issue?

2. Was the cohort recruited in an acceptable way?

3. Was the exposure accurately measured to minimise bias?

4. Was the outcome accurately measured to minimise bias?

5. Have the authors identified all important confounding factors?

6. Have they accounted the confounding factors in the design and/or analysis?

7. Was the follow up of subjects complete enough?

8. Was the follow up of subjects long enough?

9. Do you believe the results?

10. Can the results be applied to the local population?

11. Do the results of this study fit with other available evidence?

### Synthesis of results

An overview of union rate and fixation-related complications of each technique type was presented in Table 6. The studies’ data were synthesised using texts rather than statistics, lending this review’s design to a quantitative narrative synthesis. A meta-analysis was not appropriate, as initially planned, due to the lack of RCTs, data insufficiency, and heterogeneity.

**Table 6:** Overview of union rate and fixation-related complications for each fixation technique

	Screws	Plates	Staples
No. of studies	3	3	1
No. of patients	94	113	50
No. of feet	98	124	55
**Union rate (Feet)**			
Bone union in feet (%)	94.9 (93/98)	95.2 (118/124)	98.2 (54/55)
Mean time to union (days, range)	71 (40–222)	91.7 (42–183)	84 (Unknown)
**Fixation-related complications (Feet)**			
Revision surgery	2	1	-
Metalwork complications	3	2	1
Delayed union	1	-	3
Soft tissue damage	-	2	1
**Total complications, (%)**	**6.1** **(6/98)**	**4.0** **(5/124)**	**9.1** **(5/55)**

In the screw group, Patel et al. [31] utilised the MOXFQ measurement tool, obtaining a significant improvement (*p* < 0.05) in functional outcomes with mean scores of 46.4 preoperatively to 18.4 postoperatively. Similarly, Singhal et al. [32] utilised the MOXFQ measurement tool, obtaining a significant improvement (*p* < 0.05) in functional outcomes with mean scores of 49.7 preoperatively to 17.9 postoperatively. The overall mean MOXFQ score for the two studies utilising this measurement tool showed improvement from 48.1 preoperatively to 18.2 postoperatively. In contrast, Mohammed & Gadgil [30] utilised the AOFAS measurement tool, obtaining a postoperative score of 79. They did not measure the preoperative score; therefore, inferences could not be drawn to determine any improvements regarding functional outcomes preoperative to postoperatively.

In the plate group, Latif et al. [34] utilised two measurement tools to assess the functional outcomes: MOXFQ and EQ-5D. They did not disclose the overall mean for MOXFQ, although there were significant improvements (*p* < 0.05) in the walking/standing and pain domains within the MOXFQ. The numeric values were not specified. As for the EQ-5D, they presented a graph showing that the proportion of patients reporting a problem was lowered postoperatively in each of the five domains: mobility, self-care, usual activities, pain or discomfort, anxiety or depression. However, the numerical values were also not specified, and statistical analyses were not performed. Fazal et al. [33] utilised the AOFAS and FADI measurement tools to assess the functional outcomes. They obtained a significant improvement (*p* < 0.05) in mean AOFAS scores from 37.1 preoperatively to 80.7 postoperatively. Their mean FADI scores also significantly improved (*p* < 0.05) from 40.3% preoperatively to 86.9% postoperatively. Marudanayagam and Appan [35] utilised the AOFAS measurement tool, obtaining improvements in functional outcomes with mean scores of 31.0 preoperatively to 86.0 postoperatively. They did not perform any statistical analysis to determine whether there was a significant difference. The overall mean AOFAS score for the two studies that utilised this measurement tool showed improvement from 34.1 preoperatively to 83.4 postoperatively.

In the staple group, Challagundla et al. [36] utilised the FAAM ADL measurement tool, obtaining a mean score of 87% in patient satisfaction with the outcome after surgery. They did not measure the preoperative score; therefore, inferences could not be drawn to determine any improvements regarding functional outcomes preoperative to postoperatively.

## Discussion

The purpose of the present systematic review was to synthesise and compare the evidence available in the UK regarding union rate, complications, and functional outcomes following FMPJ arthrodesis. The overall union rate of feet was 95.7%, which is 5.7% higher than the typically reported union rate of 90% [12] within the broader literature. The overall complication incidence comprising revision surgery, metalwork complications, delayed union, and soft tissue damage was 5.8%. As for functional outcomes, it was difficult to synthesise the results due to vagueness in reporting, missing information, and variations in the measurement tools used.

Challagundla et al. [36], the only study using the staple technique, obtained the highest union rate of 98.2%, with a mean union time of 84 days. However, it has the highest complication incidence of 9.1%. Despite that, the staple technique received a satisfactory FAAM ADL score of 87%, which is deemed satisfactory and reinforces good clinical outcomes. Although, it is noteworthy that the FAAM tool focuses on physical functions that may be compromised by joint fusion, such as ‘coming up your toes’, which can be difficult but not impossible as neighbouring joints may compensate for the motionless FMPJ [37]. Additionally, Nixon et al. [38] could not identify a correlation between the FAAM tool and hallux rigidus grades. They also discussed a ceiling effect of the tool, as patients within their study with grade 2 or 3 hallux rigidus had perfect scores compared to those with grade 4 who had substantially lower scores, decreasing the likelihood of the FAAM tool to measure the intended domains accurately. Most FMPJ arthrodesis is indicated following symptomatic grade 3 or 4 hallux rigidus diagnosis, so this weakness must be considered when interpreting FMPJ arthrodesis functional outcome scores using the FAAM tool. Unlike the other fixation techniques, staples are less invasive and intrusive to the healthy cancellous bone and joint surface, allowing maximal bleeding bone-to-bone to contact and encourage primary bone healing to occur [39, 40]. However, Neufeld et al. [41] reported that staples provided the least stability compared to screw and plating techniques because the limbs are smooth polished metal with rounded edges, making them prone to slipping out of bone. The cohort with the three delayed unions [36] was allowed to mobilise fully weightbearing postoperatively, increasing the risk of micro-movements within the joint and leading to a delayed union. It is also noteworthy that staples were used because it was the only available fixation device in the hospital; therefore, it is arguable that the delayed unions could have been avoided if plating techniques were used since they have been shown to provide superior stability, reducing the risk of micro-movements [42]. The staples findings in this review suggest that patients should limit mobilisation and remain rested for a longer period before fully weightbearing. Even though a high union rate was achieved, this review’s data revealed staples as inferior to other techniques in terms of stability and ability to allow early weightbearing. It should, however, be viewed with scepticism because it is just one study with a small sample size [36]. Further studies are needed to determine the stability and effectiveness of staples for FMPJ arthrodesis.

The plate technique achieved an overall union rate of 95.7%, with a mean union time of 91 days. It has the lowest complication incidence of 4.0% among the three techniques for FMPJ arthrodesis. One of the included studies [33] attained a union rate of only 84.3%. The study had five non-unions, three of which were patients with rheumatoid arthritis taking anti-tumour necrosis factor medications, and two were heavy smokers, explaining the poor union rates. Patients with rheumatoid arthritis may have the dangerous liaison with osteoporosis, causing a reduction in bone mineral density (BMD) and bone remodelling [43]. Regarding smoking, Prat et al. [44] showed that tobacco use is associated with significantly higher complication rates following FMPJ arthrodesis due to nicotine increasing blood viscosity, activating vasoconstriction and reducing blood flow impeding optimal healing to occur [45]. Therefore, it is paramount to explain to patients that smoking cessation can lead to better postoperative outcomes. Nevertheless, plating techniques have been shown to provide superior stability to other techniques even in cases with poor bone stock [18, 46], allowing early full weightbearing and making them the current technique of choice in FMPJ arthrodesis for many surgeons [40, 47]. All patients who received the plating technique in this review were allowed to fully weight bear in a postoperative rigid shoe immediately. However, plating techniques do have drawbacks, including an increased risk of metalwork prominence or discomfort, longer incision line, and elimination of the feasibility to perform the recently established minimally invasive arthrodesis [40, 48]. Therefore, further innovations to the plate technique to mitigate these drawbacks are encouraged. As for functional outcomes, improvements were shown in the AOFAS-HMI and FADI scores preoperative to postoperatively. In both these tools, scores were allocated for ‘movement of joint’, which would be impossible following FMPJ arthrodesis. Therefore, the question relating to metatarsophalangeal joint motion in the AOFAS tool will have an automatic score of 0, requiring modification to be scored out of 90 instead of 100. Although widely used, several studies have criticised the tool for having sub-optimal measurement properties and questionable validity due to the lack of a sound methodological construct, discouraging its use from assessing foot-related surgical outcomes [49–51]. Alternatively, it has been recommended that where the AOFAS is used, another supplementary outcome tool should be applied that assesses patient function within society and the effect that therapeutic intervention may have on a patient’s life [52]. As for the MOXFQ, there were significant improvements in the walking/standing and pain domains [34]. The EQ-5D domains also showed improvements preoperatively to postoperatively in the bar chart, but the authors reported no descriptions [34]. The advantage of using these two tools is that it does not allocate points relating to joint movement, increasing the efficiency, reliability and validity of patients’ input in the context of FMPJ arthrodesis. While the MOXFQ was initially only validated for HAV surgery [53], it has been since shown to have good validity and reliability for a range of foot and ankle surgery [54]. However, only one study [55] attempted to show the validity of this tool in the context of hallux rigidus. Even so, only patients who underwent cheilectomy indicated by hallux rigidus grade 1 to 3 were included in the study, reducing the validity of this tool as it is unclear whether the findings can be extrapolated to patients who underwent arthrodesis where most would have a diagnosis of hallux rigidus grade 3 or 4 [2]. As outcome measures need to be validated for the population and context in which they are to be used, further validation studies are required to demonstrate the usefulness of the MOXFQ in assessing functional outcomes in FMPJ arthrodesis.

The screw technique obtained the lowest union rate of 94.9%, with a mean union time of 71 days. The overall complication rate was 6.5%, where three feet had metalwork complications, two required revision surgery, and one experienced a delayed union. The three metalwork complications derived from Patel et al.’s [31] study using the IOFIX device were due to screw head prominence at the medial cortex of the first metatarsal, which necessitated removal once union had occurred. Although, this could have been prevented through sufficient soft tissue clearance of the X-post eyelet by embedding it into the metaphyseal flare of the first metatarsal [56]. Simultaneously, the eyelet should not be positioned too deeply as it can result in the use of a shorter lag screw, causing lesser bone purchase and potential loosening of the screw and compression. Like plating techniques, all patients who received the screw technique were allowed to fully weight bear in a postoperative rigid shoe immediately. Regarding stability, two recent biomechanical studies compared the stiffness and failure load of the screw and plating techniques, finding contradicting results. Harris et al. [18] found both the screw and plate techniques having similar maximum load, but screws only exhibited approximately half the stiffness of plates. In contrast, Fuld et al. [46] demonstrated their screw group to be 64% stiffer and 46% greater in failure load than the plate group but suggested that the plate technique is preferable rather than screws in patients with poor BMD or known osteoporosis, as it may predict early failure load. Considering these and this review’s result, plating techniques have the upper edge because of the higher union rate, lower complication incidence, and better utility in those with poor bone stock. Overall, the screw technique is reliable for achieving union with minimal complications due to their relatively low profile and satisfactory functional outcomes but should be avoided in patients with inadequate bone quality.

The present systematic review was the first to evaluate the overall and individual union rates, complication incidence and functional outcomes of modern fixation techniques for FMPJ arthrodesis. Unlike the previously conducted systematic reviews [15, 57], this review was performed per PRISMA guidelines to encourage standardised writing of systematic reviews. However, the potential limitation is the absence of RCTs due to the paucity of this research design in this topic and the challenges in surgical randomisation, blinding, patient variability, and placebo control [58]. This also raises the question of whether a systematic review of RCTs can review surgical treatments. The major concern, owing to the small sample sizes within each study, is the exclusion of those lost to follow-up in their calculation in both prospective and retrospective cohort studies because this may cause an overestimation of results. It increases the likelihood of subject selection bias as those who were lost to follow-up may have a different outcome than those included for assessment. The cost of each technique was also not taken into account, which would provide invaluable information regarding cost-effectiveness. Moreover, there were variations in dissection techniques, joint surface preparation, postoperative protocols, and postoperative foot protection among studies, which were not considered due to the aim and objectives of this review. Finally, this review was a single-country study, lacking generalisability. However, our study had provided clear and comprehensive suggestions for researchers to adopt should a multi-country systematic review of FMPJ arthrodesis techniques be conducted in the future.

## Conclusions

Even though staple had the highest union rate, it is nevertheless only one outcome measure. The data from this review and biomechanical studies suggest that plating techniques are superior to other modern fixation techniques for FMPJ arthrodesis. They provide better results across several outcome measures, including reduced complication incidence, good functional outcome, stability, and early ambulation. The AOFAS tool has been condemned for having substandard measurement properties and may lack reliability in assessing functional outcomes for any foot surgery. Instead, the more reliable and validated tools – MOXFQ and EQ-5D – are recommended for use in tandem to best assess functional outcomes following FMPJ arthrodesis. Finally, researchers need to detail every aspect of their studies, including patient and feet number, follow-up times, operative technique, joint preparation, indications for surgery, pre and postoperative measurement, patient demographics and postoperative protocols to prevent biased estimates that can lead to invalid conclusions.

## Supplementary Information

**Additional file 1.**

**Additional file 2.**

**Additional file 3.**

## Abbreviations

AOFAS-HMI: American Orthopaedic Foot and Ankle Society-Hallux Metatarsophalangeal Interphalangeal
CASP: Critical Appraisal Skills Programme
EQ-5D: EuroQol–5 Dimensional
FAAM ADL: Foot and Ankle Mobility Measure (Activities of Daily Living)
FADI: Foot and Ankle Disability Index
FMPJ: first metatarsophalangeal joint
HAV: hallux abducto valgus
IOFIX: Intra-Osseous FIXation
MOXFQ: Manchester-Oxford Foot Questionnaire
NHS: National Health Service
PRISMA: Preferred Reporting Items for Systematic Reviews and Meta-Analyses
RCT: Randomised Controlled Trial
UK: United Kingdom
