# Supplementary material for: First metatarsophalangeal joint arthrodesis/fusion: a systematic review of modern fixation techniques
Source: J Foot Ankle Res. 2022 Apr 26;15:30. doi: 10.1186/s13047-022-00540-9 (PMC9040205; doi:10.1186/s13047-022-00540-9)
Supplement: Supplementary file 2 — Additional file 2. [file 13047_2022_540_MOESM2_ESM.docx]

**Additional file 2: Search strategies for Cochrane Library, PubMed, EBSCO CINAHL, and Google Scholar.**

Cochrane Library search terms.

| ID | Search |
| --- | --- |
| #1 | exp Arthrodesis/ |
| #2 | "arthrodesis" OR "ankylosis" OR "fusion" OR "arthrodeses" |
| #3 | #1 OR #2 |
| #4 | Exp Metatarsophalangeal Joint/ |
| #5 | "first metatarsophalangeal joint" OR "first metatarsal-phalangeal joint" OR "hallux metatarsophalangeal joint" OR “hallux metatarsal-phalangeal joint” OR "MTPJ" OR "1MTP*" OR "FMTP*" OR "FMPJ" OR "1MPJ" OR "HMPJ" OR "HMTP*" OR "MPJ" OR “HMP” |
| #6 | #4 OR #5 |
| #7 | exp Surgical Stapling/ |
| #8 | exp Bone Screws/ |
| #9 | exp Bone Plates/ |
| #10 | "screw*" OR "staple*" OR "plate*" |
| #11 | #7 OR #8 OR #9 OR #10 |
| #12 | exp United Kingdom/ |
| #13 | "united kingdom" OR "UK" OR "great britain" OR "brit*" OR "GB" OR "engl*" OR "scot*" OR "northern ireland" OR "irish" OR "wales" OR "welsh" |
| #14 | #12 OR #13 |
| #15 | #3 AND #6 AND #11 AND #14 |

PubMed search terms.

| ((("arthrodesed"[All Fields] OR "arthrodesing"[All Fields] OR "arthrodesis"[MeSH Terms] OR "arthrodesis"[All Fields] OR "arthrodese"[All Fields] OR "arthrodeses"[All Fields] OR ("fusion"[All Fields] OR "fusions"[All Fields]) OR ("ankylose"[All Fields] OR "ankylosed"[All Fields] OR "ankylosis"[MeSH Terms] OR "ankylosis"[All Fields] OR "ankyloses"[All Fields]) OR ("arthrodesed"[All Fields] OR "arthrodesing"[All Fields] OR "arthrodesis"[MeSH Terms] OR "arthrodesis"[All Fields] OR "arthrodese"[All Fields] OR "arthrodeses"[All Fields])) AND ("humans"[MeSH Terms] AND "english"[Language]) AND (((("first"[All Fields] OR "firsts"[All Fields]) AND ("metatarsophalangeal joint"[MeSH Terms] OR ("metatarsophalangeal"[All Fields] AND "joint"[All Fields]) OR "metatarsophalangeal joint"[All Fields])) OR (("first"[All Fields] OR "firsts"[All Fields]) AND ("metatarsophalangeal joint"[MeSH Terms] OR ("metatarsophalangeal"[All Fields] AND "joint"[All Fields]) OR "metatarsophalangeal joint"[All Fields] OR ("metatarsal"[All Fields] AND "phalangeal"[All Fields] AND "joint"[All Fields]) OR "metatarsal phalangeal joint"[All Fields])) OR (("hallux"[MeSH Terms] OR "hallux"[All Fields]) AND ("metatarsophalangeal joint"[MeSH Terms] OR ("metatarsophalangeal"[All Fields] AND "joint"[All Fields]) OR "metatarsophalangeal joint"[All Fields])) OR (("hallux"[MeSH Terms] OR "hallux"[All Fields]) AND ("metatarsophalangeal joint"[MeSH Terms] OR ("metatarsophalangeal"[All Fields] AND "joint"[All Fields]) OR "metatarsophalangeal joint"[All Fields] OR ("metatarsal"[All Fields] AND "phalangeal"[All Fields] AND "joint"[All Fields]) OR "metatarsal phalangeal joint"[All Fields])) OR "MTPJ"[All Fields] OR "1mtp*"[All Fields] OR "fmtp*"[All Fields] OR "FMPJ"[All Fields] OR "1MPJ"[All Fields] OR "hmtp*"[All Fields] OR "MPJ"[All Fields] OR "HMP"[All Fields]) AND ("humans"[MeSH Terms] AND "english"[Language])) AND (("bone screws"[MeSH Terms] OR ("bone"[All Fields] AND "screws"[All Fields]) OR "bone screws"[All Fields] OR "screw"[All Fields] OR "screw s"[All Fields] OR "screwed"[All Fields] OR "screwing"[All Fields] OR "screws"[All Fields] OR ("stapl"[All Fields] OR "stapled"[All Fields] OR "stapling"[All Fields] OR "sutures"[MeSH Terms] OR "sutures"[All Fields] OR "staple"[All Fields] OR "staples"[All Fields]) OR ("bone plates"[MeSH Terms] OR ("bone"[All Fields] AND "plates"[All Fields]) OR "bone plates"[All Fields] OR "plate"[All Fields] OR "plate s"[All Fields] OR "plated"[All Fields] OR "plates"[All Fields] OR "plating"[All Fields] OR "platings"[All Fields])) AND ("humans"[MeSH Terms] AND "english"[Language])) AND (("united kingdom"[MeSH Terms] OR ("united"[All Fields] AND "kingdom"[All Fields]) OR "united kingdom"[All Fields] OR "uk"[All Fields] OR "gb"[All Fields] OR ("britain"[All Fields] OR "britain s"[All Fields] OR "britains"[All Fields]) OR "british"[All Fields] OR ("england"[MeSH Terms] OR "england"[All Fields] OR "england s"[All Fields] OR "englands"[All Fields]) OR "english"[All Fields] OR ("scotland"[MeSH Terms] OR "scotland"[All Fields] OR "scotland s"[All Fields]) OR "scottish"[All Fields] OR ("northern ireland"[MeSH Terms] OR ("northern"[All Fields] AND "ireland"[All Fields]) OR "northern ireland"[All Fields]) OR "irish"[All Fields] OR ("wales"[MeSH Terms] OR "wales"[All Fields] OR "wales s"[All Fields]) OR "welsh"[All Fields]) AND ("humans"[MeSH Terms] AND "english"[Language]))) NOT ("lapidus"[All Fields] AND ("humans"[MeSH Terms] AND "english"[Language]))) AND ((humans[Filter]) AND (english[Filter]) AND (2010:2021[pdat])) |
| --- |

EBSCO CINAHL search terms.

| (arthrodesis OR ankylosis OR fusion OR arthrodeses)  AND  (first metatarsophalangeal joint OR first metatarsal-phalangeal joint OR hallux metatarsophalangeal joint OR hallux metatarsal-phalangeal joint OR MTPJ OR 1MTP* OR FMTP* OR FMPJ OR 1MPJ OR HMPJ OR HMTP* OR MPJ OR HMP)  AND  (screw* OR staple* OR plate*)  AND  (united kingdom OR UK OR great britain OR brit* OR GB OR engl* OR scot* OR northern ireland OR irish OR wales OR welsh) |
| --- |

Google Scholar search terms.

| First metatarsophalangeal joint arthrodesis using screw, plate or staple in the United Kingdom |
| --- |
