# Supplementary material for: First metatarsophalangeal joint arthrodesis/fusion: a systematic review of modern fixation techniques
Source: J Foot Ankle Res. 2022 Apr 26;15:30. doi: 10.1186/s13047-022-00540-9 (PMC9040205; doi:10.1186/s13047-022-00540-9)
Supplement: Supplementary file 3 — Additional file 3. [file 13047_2022_540_MOESM3_ESM.docx]

**Additional file 2: Reasons for exclusion of full-text reports**

| **Study** | **Title** | **Reason for exclusion at full-text screening.** |
| --- | --- | --- |
| Dayton et al. (2019) | Progression of Healing on Serial Radiographs Following First Ray Arthrodesis in the Foot Using a Biplanar Plating Technique Without Compression. | Non-UK study. |
| Chan et al. (2019) | Failure of Fixation With Nickel-Titanium Staples in First Metatarsophalangeal Arthrodesis With Hallux Valgus Deformity. | Non-UK study. |
| Asif et al. (2018) | A Consecutive Case Series of 166 First Metatarsophalangeal Joint Fusions Using a Combination of Cup and Cone Reamers and Crossed Cannulated Screws. | Functional outcome not assessed. |
| Marsland et al. (2016) | Fusion of the First Metatarsophalangeal Joint: Precontoured or Straight Plate? | Union rate, complication rate and functional outcome not assessed. |
| Baumhauer et al. (2016) | Prospective, Randomized, Multi-centred Clinical Trial Assessing Safety and Efficacy of a Synthetic Cartilage Implant Versus First Metatarsophalangeal Arthrodesis in Advanced Hallux Rigidus | Multi-centred study with participants from UK and Canada. Unable to extract data from the UK sample as they were indistinguishable. |
| Korim & Allen (2015) | Effect of pathology on union of first metatarsophalangeal joint arthrodesis | Functional outcome not assessed. |
| Bass & Sirikonda (2015) | 1st metatarsophalangeal joint fusion: A comparison of non-union and gender differences between locking and non-locking plating systems | Functional outcome not assessed. |
| Shah et al. (2012) | Arthrodesis of the first metatarsophalangeal joint: comparison of three techniques | Functional outcome not assessed. |
